# Asymmetric Synthesis and Evaluation of Danshensu-Cysteine Conjugates as Novel Potential Anti-Apoptotic Drug Candidates

**DOI:** 10.3390/ijms16010628

**Published:** 2014-12-29

**Authors:** Li-Long Pan, Jie Wang, Yao-Ling Jia, Hong-Ming Zheng, Yang Wang, Yi-Zhun Zhu

**Affiliations:** 1 Shanghai Key Laboratory of Bioactive Small Molecules, Department of Pharmacology, School of Pharmacy, Fudan University, Shanghai 201203, China; E-Mails: panlilong@fudan.edu.cn (L.-L.P.); zhenghm1985born@hotmail.com (H.-M.Z.); 2 Department of Medicinal Chemistry, School of Pharmacy, Fudan University, Shanghai 201203, China; E-Mails: wangjie@fudan.edu.cn (J.W.); jiayaoling211@163.com (Y.-L.J.)

**Keywords:** Danshensu derivative, apoptosis, asymmetric synthesis, endothelial cells

## Abstract

We have previously reported that the danshensu-cysteine conjugate *N*-((*R*)-3-benzylthio-1-methoxy-1-oxo-2-propanyl)-2-acetoxy-3-(3,4-diacetoxyphenyl) propanamide (DSC) is a potent anti-oxidative and anti-apoptotic agent. Herein, we further design and asymmetrically synthesize two diastereoisomers of DSC and explore their potential bioactivities. Our results show that DSC and its two diastereoisomers exert similar protective effects in hydrogen peroxide (H_2_O_2_)-induced cellular injury in SH-SY5Y cells, as evidenced by the increase of cell viability, superoxide dismutase (SOD), and reduced glutathione (GSH) activity, and glutathione peroxidase (GPx) expression, and the decrease of cellular morphological changes and nuclear condensation, lactate dehydrogenase (LDH) release, and malondialdehyde (MDA) production. In H_2_O_2_-stimulated human umbilical vein endothelial cells (HUVEC), DSC concentration-dependently attenuates H_2_O_2_-induced cell death, LDH release, mitochondrial membrane potential collapse, and modulates the expression of apoptosis-related proteins (Bcl-2, Bax, caspase-3, and caspase-9). Our results provide strong evidence that DSC and its two diastereoisomers have similar anti-oxidative activity and that DSC exerts significant vascular-protective effects, at least in part, through inhibition of apoptosis and modulation of endogenous antioxidant enzymes.

## 1. Introduction

Oxidative stress occurs when reactive oxygen species (ROS) are generated in excess through the reduction of oxygen, which can destroy the physiological function of cellular proteins, lipids, nucleic acids, and other macromolecular substances [1]. Oxidative stress is implicated in the pathogenesis of neurodegenerative and cardiovascular disorders, such as dysautonomia, Alzheimer’s disease, atherosclerosis, peripheral artery disease, myocardial ischemia, and so on [2,3,4,5]. Indeed, oxidative stress, generated by excessive amounts of ROS including hydrogen peroxide (H_2_O_2_), causes cell apoptosis and plays an essential role in the pathogenesis of neurodegenerative and cardiovascular disorders [5,6,7]. Therefore, pharmacological approaches for intervening in oxidative stress may provide therapeutic intervention strategies for neurodegenerative and cardiovascular disorders.

**Figure 1:**
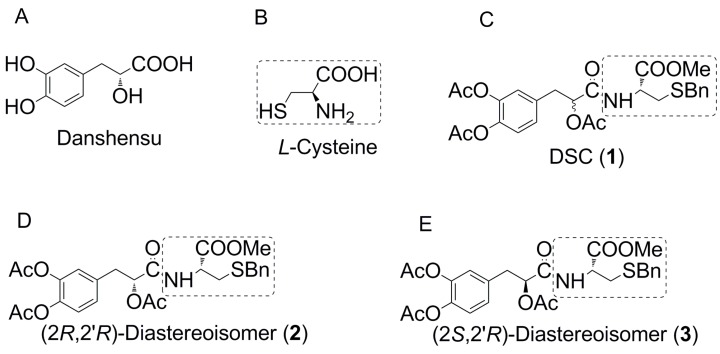
The structures of Danshensu, l-cysteine and their conjugated derivatives (**1**–**3**). (**A**) Danshensu; (**B**) l-Cysteine; (**C**) *N*-((*R*)-3-benzylthio-1-methoxy-1-oxo-2-propanyl)-2-acetoxy-3-(3,4-diacetoxyphenyl) propanamide (DSC); (**D**) (2*R*,2'*R*)-diastereoisomer of DSC; (**E**) (2*S*,2'*R*)-diastereoisomer of DSC.

*Salvia miltiorrhiza* Bunge (Danshen in Chinese) or *S. miltiorrhiza*, a perennial herbal plant of the Labiatae family, is widely used for the treatment of coronary heart disease and other cardiovascular disorders, blood circulation diseases, and menstrual disorders in Eastern countries for hundreds of years [8,9]. Danshensu ((*R*)-3-(3,4-dihydroxyphenyl)-2-hydroxypropanoic acid, Figure 1A), a hydrophilic bioactive component of Danshen, attracts considerable interest due to its salubrious biological activities, such as coronary artery dilatation, microcirculation improvement, myocardial protection and anti-platelet aggregation [6,10]. However, the chemical instability of phenolic hydroxyl groups of danshensu results in poor cellular permeability, bioavailability, lipophilicity and low pharmacological potency, which limits its further therapeutic development for clinical use [11]. Many synthetic Danshensu compounds with modified groups have emerged, laying the foundation of exploring and improving the biological activity of Danshensu.

In our previous studies, an array of Danshensu derivatives were synthesized by combination with l-cysteine (Figure 1B) through an appropriate bond in the light of the guidance of medicinal chemical hybridization [12]. Among them, DSC (1,*N*-((*R*)-3-benzylthio-1-methoxy-1-oxo-2-propanyl)-2-acetoxy-3-(3,4-diacetoxyphenyl) propanamide, Figure 1C), a novel Danshensu-cysteine conjugate, has aroused considerable interest due to its various biological activities, such as anti-oxidative [6] and anti-inflammatory [10] capacities. In the present study, we thus hypothesize that the diastereoisomers of DSC also possess antioxidant activities. Therefore two diastereoisomers **2** and **3** (Figure 1D,E) of DSC were further obtained by asymmetric synthesis for antioxidant screening, and we herein present the synthesis and anti-oxidative and anti-apoptotic activities of Danshensu-cysteine conjugates in H_2_O_2_-induced cellular damage in SH-SY5Y cells and human umbilical vein endothelial cells (HUVEC).

## 2. Results and Discussion

### 2.1. The Asymmetric Synthesis of Danshensu-Cysteine Conjugates

**Figure 2:**
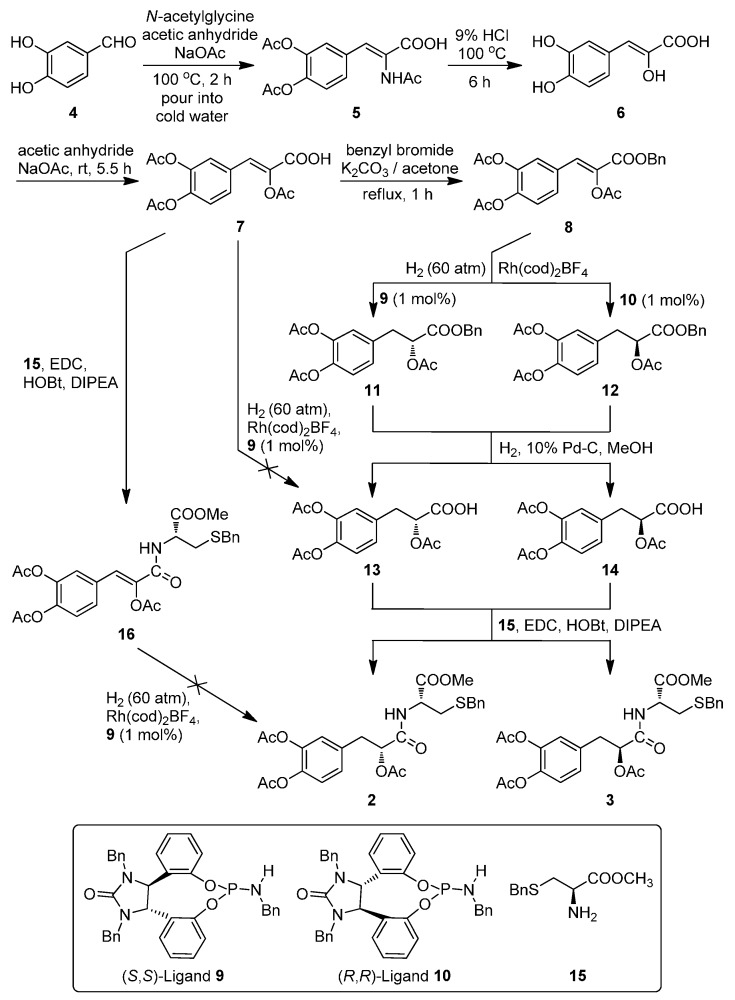
The synthesis of target diastereoisomers **2** and **3**.

As outlined in Figure 2, the designed conjugates were synthesized efficiently using 3,4-dihydroxybenzaldehyde (**4**) as the starting material. The key intermediate **7** and l-cysteine derivatives **15** were synthesized according to the reported procedures [12,13]. The condensation of **4** with *N*-acetylglycine followed by hydrolysis in hydrochloric acid and protection of the hydroxyl groups in acetic anhydride afforded **7** in 33% total yield. Esterization of **7** with benzyl bromide in the presence of K_2_CO_3_ in acetone provided the thoroughly protected compound **8** in 90% yield. The asymmetric hydrogenation of **8** using **9**/**10** as the chiral ligand and [Rh(cod)_2_]BF_4_ as the catalyst precursor gave excellent enantioselectivities (>97% enantiomeric excess) and good yields (>89%) of compounds **11**/**12** respectively under the modified conditions of the literature procedures [14]. The deprotection of **11**/**12** by hydrogenolysis under the catalysis of 10% Pd-C in methanol attained (*R*)/(*S*)-2-acetoxy-3-(3,4-diacetoxyphenyl) propanoic acid (**13**/**14**) in >95% yields, which were then subjected to the condensation with *S*-benzyl-l-cysteine methyl ester (**15**) in the presence of 1-hydroxybenzotriazole (HOBt), 1-(3-dimethylaminopropyl)-3-ethylcarbodiimide hydrochloride (EDCI) and diisopropylethylamine (DIPEA) in CH_2_Cl_2_, affording the target amide conjugates **2**/**3** in 79% and 81% yields respectively.

Another synthetic route to **2**/**3** was also tried *via* the direct asymmetric hydrogenation of **7** to give **13** or condensation of **7** with **15** followed by asymmetric hydrogenation of **16**, but both of the asymmetric hydrogenation of **7**/**16** failed which may be attributed to the reactive hydrogen in the carboxy group of **7** and the sulphur atom or steric-hindrance in **16**.

#### 2.1.1. (*Z*)-2-Acetoxy-3-(3,4-diacetoxyphenyl)acrylic Acid Benzyl Ester (**8**)

(*Z*)-2-Acetoxy-3-(3,4-diacetoxyphenyl)acrylic acid (**7**) was prepared by using aldehyde **4** as the starting material according to the method in the literature [13]. To a solution of a mixture of **7** (1.5 g, 4.65 mmol) and anhydrous K_2_CO_3_ (1.28 g, 9.31 mmol) in acetone (30 mL) was dropped benzyl bromide (1.5 mL, 12.6 mmol). The mixture was refluxed for 7 h, then filtered and concentrated. The residue was purified by silica gel column chromatography (eluent:PE-EtOAc, 6:1~3:1), providing 1.72 g of **8** as a white solid in 90% yield, mp 79–82 °C. ^1^H NMR (400 MHz, CDCl_3_) δ 2.28 (s, 9H, –OCOCH_3_), 5.28 (s, 2H, –CH_2_Ph), 7.21 (d, *J* = 8.6 Hz, 1H), 7.27 (d, *J* = 5.5 Hz, 1H), 7.35–7.40 (m, 5H), 7.43 (d, *J* = 8.8 Hz, 1H), 7.47 (s, 1H). ESI-MS *m*/*z* (%): 435.2 (M + Na^+^, 100). High Resolution Mass Spectrum (HRMS) calculated mass for C_22_H_20_O_8_Na [M + Na^+^] 435.1056, found 435.1050.

#### 2.1.2. (*R*)/(*S*)-2-Acetoxy-3-(3,4-diacetoxyphenyl)propanoic Acid Benzyl Ester (**11**/**12**)

[Rh(cod)_2_]BF_4_ (2.0 mg, 0.005 mmol), ligand **9** (6 mg, 0.011 mmol) in CH_2_Cl_2_ (1 mL) were dissolved under nitrogen and the solution was stirred at room temperature for 10 min. The substrate **8** (206 mg, 0.5 mmol) in CH_2_Cl_2_ (2 mL) was added to the above catalyst solution. The mixture was then transferred to a stainless steel autoclave under nitrogen atmosphere, and then sealed. After purging with hydrogen three times, final H_2_ pressure was adjusted to 60 atm. After stirring at room temperature for 24 h, H_2_ was released. After removal of the solvent under the reduced pressure, the residue of the product was passed through a pad of Celite and purified by silica gel column chromatography (eluent:PE-EtOAc, 5:1~3:1), providing 184 mg of **11** as a colorless oil in 89% yield and 97% *ee*, $\left[{\text{α}}_{\text{D}}^{20}\right]$ = +4.8° (c = 1.0 mg/mL, CHCl_3_). The enantiomeric excess was determined by HPLC on Chiralcel OJ column, hexane:isopropanol = 95:5; flow rate = 1.0 mL/min; UV detection at λ = 230 nm; *t*_R_ = 16.1 min (minor), 19.8 min (major), respectively. Using **10** as chiral ligand, 186 mg of product **12** could be obtained as a colorless oil in 90% yield and 98% *ee*, $\left[{\text{α}}_{\text{D}}^{20}\right]$ = −4.8° (c = 1.0 mg/mL, CHCl_3_). ^1^H NMR (400 MHz, CDCl_3_), δ 2.1 (s, 3H, –OCOCH_3_), 2.29 (s, 6H, –OCOCH_3_), 3.06–3.19 (m, 2H, –CH_2_Ar), 5.17 (s, 2H, –CH_2_Ph), 5.20–5.25 (m, 1H, –CHOCOCH_3_), 7.05–7.11 (m, 3H, *–*Ar), 7.27–7.39 (m, 5H, –Ph). ESI-MS *m*/*z* (%): 437.3 (M + Na^+^, 100). HRMS calculated mass for C_22_H_22_O_8_Na [M + Na^+^] 437.1212, found 437.1207.

#### 2.1.3. (*R*)/(*S*)-2-Acetoxy-3-(3,4-diacetoxyphenyl)propanoic Acid (**13**/**14**)

Compounds **11**/**12** (200 mg, 0.48 mmol) were deprotected by hydrogenolysis under the catalysis of 10% Pd-C (200 mg) at atmospheric pressure in MeOH (5 mL) for 24 h. The mixture was filtered and concentrated, and the residue was purified by silica gel column chromatography (eluent:PE-EtOAc, 10:1~5:1) to give **13** (149 mg)/**14** (148 mg) as sticky colorless oils in 96%/95% yields respectively. ^1^H NMR (400 MHz, CDCl_3_), δ 2.17 (s, 3H, –OCOCH_3_), 2.27 (s, 6H, –OCOCH_3_), 3.12–3.18 (m, 2H, –CH_2_–), 5.21 (br, 1H, –COOH), 7.09–7.12 (m, 3H, Ph–H).

#### 2.1.4. (*R*)-*N*-((*R*)-3-Benzylthio-1-methoxy-1-oxo-2-propanyl)-2-acetoxy-3-(3,4-diacetoxyphenyl)propanamide (**2**) and (*S*)-*N*-((*R*)-3-Benzylthio-1-methoxy-1-oxo-2-propanyl)-2-acetoxy-3-(3,4-diacetoxyphenyl)propanamide (**3**)

To a solution of carboxylic acid **13**/**14** (162 mg, 0.5 mmol) in CH_2_Cl_2_ (8 mL) was added HOBt (82 mg, 0.6 mmol) and EDCI (116 mg, 0.6 mmol). The mixture was stirred at room temperature for 15 min, then methyl *S*-benzyl-l-cysteine hydrochloride (**15**) (244 mg, 0.55 mmol) followed by diisopropylethylamine (0.1 mL, 0.6 mmol) were added. After 2 h, The mixture was filtered and concentrated, and the residue was purified by silica-gel column chromatography to give the corresponding amide conjugates **2** (210 mg)/**3** (215 mg) as white viscous oils in 79%/81% yields and 97%/73% diastereoisomeric excess, respectively. Compound **2**: $\left[{\text{α}}_{\text{D}}^{20}\right]$ = +12.8° (c = 1.0 mg/mL, CHCl_3_). ^1^H NMR (400 MHz, CDCl_3_) δ 2.11, 2.13 (s, s, 3H, –OCOCH_3_), 2.25–2.27 (m, 6H, –OCOCH_3_), 2.78–2.87 (m, 2H, –CH_2_SBn), 3.11–3.21 (m, 2H, ArC***H***_2_CH–), 3.65 (s, 2H, –SCH_2_Ph), 3.74 (s, 3H, –COOCH_3_), 4.71–4.75 (m, 1H, –NCH–), 5.36–5.40 (m, 1H, ArCH_2_C***H***–), 6.75 (t, *J* = 7.9 Hz, 1H, –NH–), 7.05–7.09 (m, 3H, Ph–H), 7.24–7.33 (m, 5H, Ph–H). ^13^C NMR (100 MHz, CDCl_3_), δ 170.8, 169.5, 168.8, 168.2, 168.1, 141.8, 141.0, 137.5, 134.5, 129.0, 128.9, 128.6, 127.6, 127.3, 124.7, 123.3, 73.7, 52.7, 51.2, 36.9, 36.4, 33.3, 33.0, 20.8, 20.6. ESI-MS *m*/*z* (%): 554.8 (M + Na^+^, 100). HRMS calculated mass for C_26_H_30_NO_9_S [M + H^+^] 532.1636, found 532.1633. Compound **3**: $\left[{\text{α}}_{\text{D}}^{20}\right]$ = +8.9° (c = 1.0 mg/mL, CHCl_3_).

### 2.2. Effects of Danshensu-Cysteine Conjugates (DSC (1,N-((R)-3-Benzylthio-1-methoxy-1-oxo-2-propanyl)-2-acetoxy-3-(3,4-diacetoxyphenyl)), **2** and **3**) on H_2_O_2_-Induced Cellular Injury in SH-SY5Y Cells

**Figure 3:**
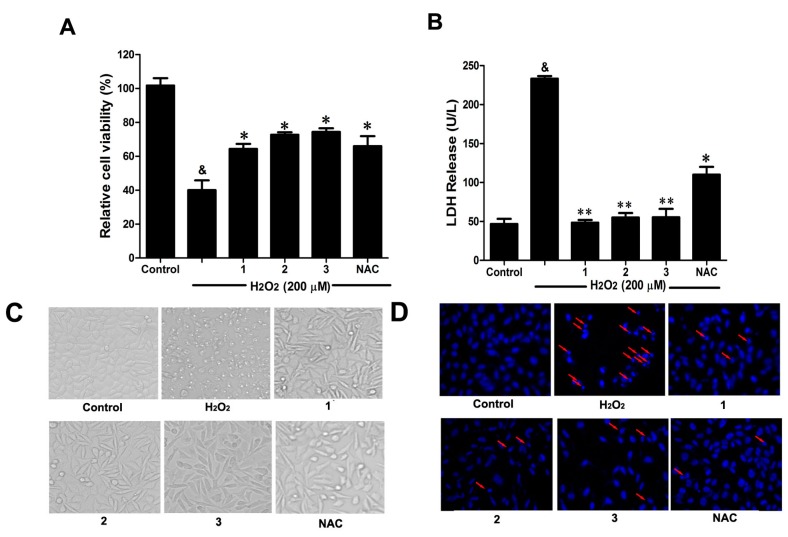
Effects of Danshensu-cysteine conjugates on H_2_O_2_-induced cellular injury in SH-SY5Y cells. SH-SY5Y cells were incubated with Danshensu-cysteine conjugates (DSC, **2** and **3**) (all at 50 μM) or *N*-acetyl-l-cysteine (NAC, 5 mM) for 4 h, then stimulated with H_2_O_2_ (200 μM) for 12 h, the cell viability (**A**) and lactate dehydrogenase (LDH) (**B**) release were measured, respectively. Data shown are means ± SEM, ^&^ *p* < 0.05 compared with unstimulated cells, * *p* < 0.05, ** *p* < 0.01 compared with H_2_O_2_-stimulated cells. Data were from at least three independent experiments, each performed in duplicate. Representative picture shows cell morphology (**C**) and nuclear morphology (**D**) detected by microscope or fluorescence microscope (magnification, 200×), respectively.

Consistent with our previous study [6], H_2_O_2_ (200 μM) stimulation for 12 h significantly decreased cell viability, accompanied by serious lactate dehydrogenase (LDH) release (Figure 3A,B). However, pretreatment with the three Danshensu-cysteine conjugates (DSC, **2** and **3**) (all at 50 μM), showed similar protective effects on H_2_O_2_-induced cell death and LDH release in SH-SY5Y cells. Meanwhile, we also checked the effects of these three compounds on H_2_O_2_-induced changes of cellular morphology and nuclear morphological changes. As shown in Figure 3C,D, pretreatment with the three derivatives (DSC, **2** and **3**) (all at 50 μM) also showed similar protective effects on H_2_O_2_-induced cellular morphological changes and nuclear morphological changes. As a positive control, *N*-acetyl-l-cysteine (NAC, 5 mM) could markedly attenuate H_2_O_2_-mediated cellular events mentioned above. Collectively, DSC and its two diastereoisomers (**2** and **3**) showed similar protective effects in H_2_O_2_-induced cellular injury in SH-SY5Y cells.

### 2.3. Effects of Danshensu-Cysteine Conjugates on Intercellular Anti-Oxidative Capacity in H_2_O_2_-Stimulated SH-SY5Y Cells

**Figure 4:**
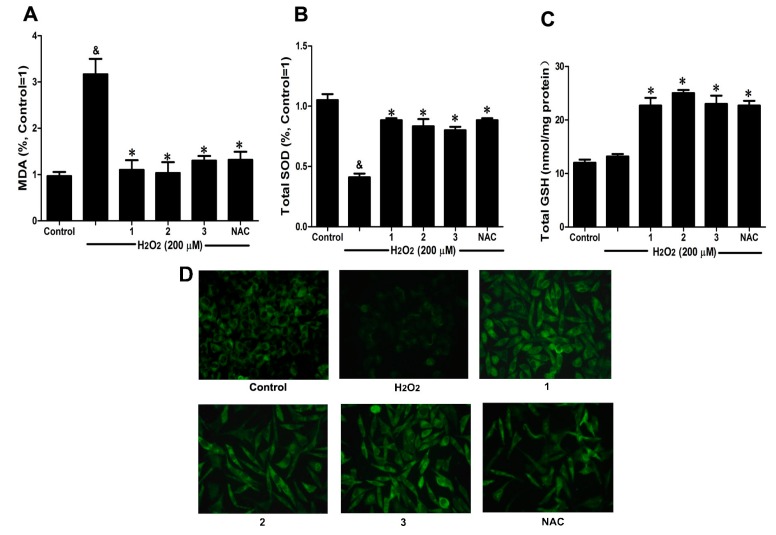
Effects of Danshensu-cysteine conjugates on intercellular anti-oxidative capacity in H_2_O_2_-stimulated SH-SY5Y cells. SH-SY5Y cells were incubated with Danshensu-cysteine conjugates (all at 50 μM) or NAC (5 mM) for 4 h, then stimulated with H_2_O_2_ (200 μM) for 12 h; the malondialdehyde (MDA) (**A**) production, superoxide dismutase (SOD) (**B**) and reduced glutathione (GSH) (**C**) activities were measured, respectively. Data shown are means ± SEM, ^&^ *p* < 0.05 compared with unstimulated cells, * *p* < 0.05 compared with H_2_O_2_-stimulated cells. Data were from at least three independent experiments, each performed in duplicate; (**D**) Representative pictures showing glutathione peroxidase (GPx) expression by immunofluorescent staining (magnification, 200×).

To further elucidate the protective effects of Danshensu-cysteine conjugates on H_2_O_2_-induced cellular injury, malondialdehyde (MDA) production, intracellular total superoxide dismutase (SOD) and reduced glutathione (GSH) activity as well as glutathione peroxidase (GPx) expression were measured by commercial kits and immunofluorescence, respectively. As shown in Figure 4A,B, H_2_O_2_ stimulation significantly decreased intercellular anti-oxidative capacity as evidenced by the increase of MDA production and reduction of SOD activity, which were evidently ameliorated by pretreatment with Danshensu-cysteine conjugates (DSC, **2** and **3**) (all at 50 μM) or NAC (5 mM). Although H_2_O_2_ stimulation did not decrease GSH activity, incubation with the three derivatives (DSC, **2** and **3**) (all at 50 μM) or NAC (5 mM) significantly increased GSH activity in H_2_O_2_-stimulated cells (Figure 4C). In addition, three conjugates (DSC, **2** and **3**) (all at 50 μM) or NAC (5 mM) also evidently blocked H_2_O_2_-induced decrease of GPx expression in SH-SY5Y cells as detected by immunofluorescent staining (Figure 4D). These data strongly suggested that DSC and its two diastereoisomers (**2** and **3**) could improve intercellular anti-oxidative capacity in H_2_O_2_-stimulated SH-SY5Y cells, but these effects were not statistically significant between the three compounds at the dosage used in this study.

### 2.4. DSC Attenuated H_2_O_2_-Induced Cell Damage in Human Umbilical Vein Endothelial Cells (HUVEC)

**Figure 5:**
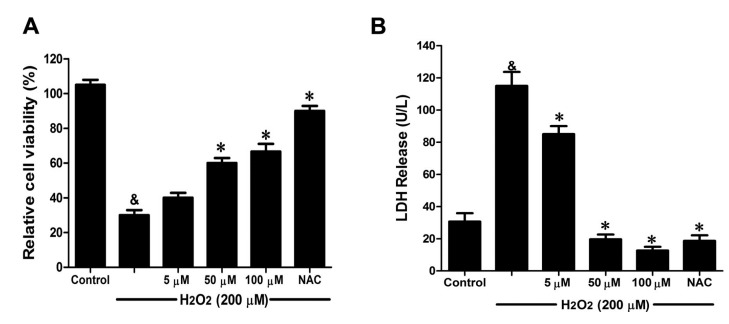
The danshensu-cysteine conjugate *N*-((*R*)-3-benzylthio-1-methoxy-1-oxo-2-propanyl)-2-acetoxy-3-(3,4-diacetoxyphenyl) propanamide (DSC) attenuated H_2_O_2_-induced cell damage in human umbilical vein endothelial cells (HUVEC). HUVEC were incubated with indicated concentrations of DSC or NAC for 4 h, then stimulated with H_2_O_2_ (200 μM) for 12 h; the cell viability (**A**) and LDH (**B**) release were measured, respectively. Data shown are means ± SEM, ^&^ *p* < 0.05 compared with unstimulated cells, * *p* < 0.05 compared with H_2_O_2_-stimulated cells. Data were from at least three independent experiments, each performed in duplicate.

Oxidative stress is one of the mechanisms that cause widespread endothelial dysfunction in cardiovascular diseases and disorders [15]. To examine the endothelial protective effects of Danshensu-cysteine conjugates on oxidative stress-induced cytotoxicity, HUVEC were conditioned with Danshensu-cysteine conjugates for 4 h prior to H_2_O_2_ stimulation. Taking into account the difficulty of chemical synthesis, only DSC was chosen to study the protective effects of Danshensu-cysteine conjugates on H_2_O_2_-induced cellular injury. As shown in Figure 5A, H_2_O_2_ (200 μM) stimulation for 12 h significantly decreased cell viability, which was attenuated by DSC (5–100 μM) in a concentration-dependent manner. Meanwhile, DSC (5–100 μM) also decreased H_2_O_2_-induced LDH release in a concentration dependent manner (Figure 5B). In addition, DSC (5–200 μM) treatment for 24 h did not affect cell viability of HUVEC as checked by MTT assay (data not shown). As a positive control, NAC also markedly reduced H_2_O_2_-induced cellular damage in HUVEC (Figure 5A,B).

### 2.5. DSC Improved Anti-Oxidative Capacity in H_2_O_2_-Stimulated HUVEC

**Figure 6:**
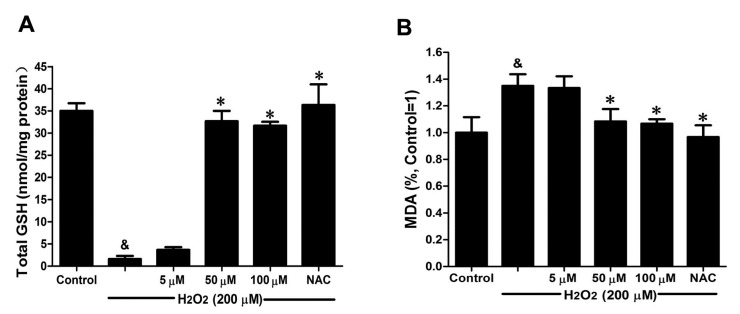
DSC improved anti-oxidative capacity in H_2_O_2_-stimulated HUVEC. HUVEC were incubated with indicated concentrations of DSC or NAC for 4 h, then stimulated with H_2_O_2_ (200 μM) for 12 h; the GSH activity (**A**) and MDA (**B**) production were measured, respectively. Data shown are means ± SEM, ^&^ *p* < 0.05 compared with unstimulated cells, * *p* < 0.05 compared with H_2_O_2_-stimulated cells. Data were from at least three independent experiments, each performed in duplicate.

GSH activity and MDA production were chosen to evaluate the effects of DSC on anti-oxidative capacity in H_2_O_2_-stimulated HUVEC. As shown in Figure 6A, H_2_O_2_ (200 μM) stimulation for 12 h markedly decreased GSH activity (*p* < 0.05), which was reversed by DSC pretreatment in a concentration-dependent manner. Meanwhile, a significant increase in MDA release was observed after stimulation with H_2_O_2_ for 12 h (*p* < 0.05), reflecting a decrease in anti-oxidative ability. However, pretreatment with DSC also resulted in a decrease of H_2_O_2_-induced MDA levels in HUVEC (Figure 6B). Meanwhile, NAC markedly enhanced intercellular anti-oxidative capacity in H_2_O_2_-stimulated HUVEC.

### 2.6. DSC Inhibited H_2_O_2_-Induced Mitochondrial Membrane Potential (ΔΨ_m_) Loss and Apoptosis in HUVEC

**Figure 7:**
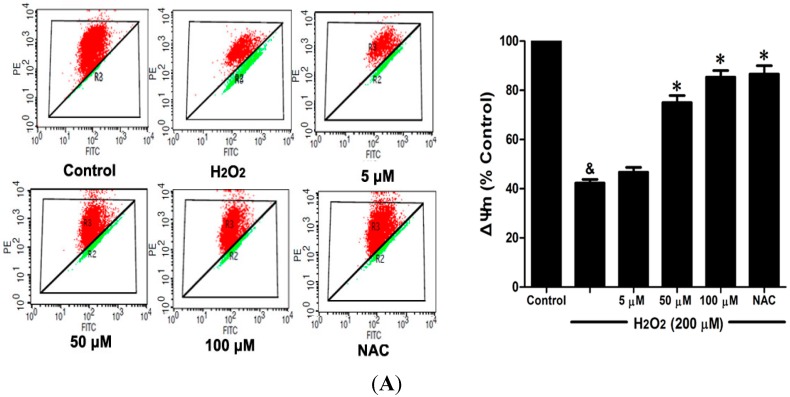

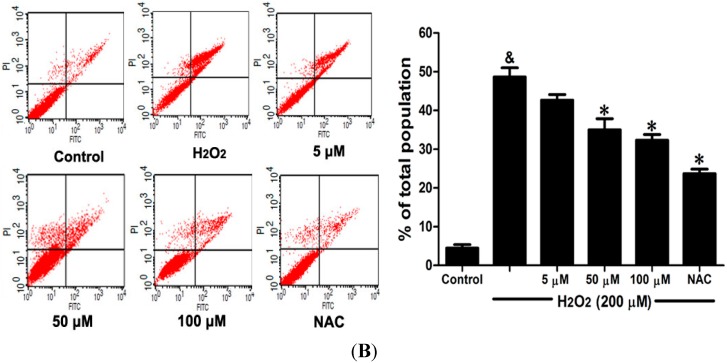
DSC inhibited H_2_O_2_-induced mitochondrial membrane potential (ΔΨ_m_) loss and apoptosis in HUVEC. HUVEC were incubated with indicated concentrations of DSC or NAC for 4 h, then stimulated with H_2_O_2_ (200 μM) for 2 or 12 h; the ΔΨ_m_ (**A**) and apoptosis (**B**) were measured by flow cytometry, respectively. Data shown are means ± SEM, ^&^ *p* < 0.05 compared with unstimulated cells, * *p* < 0.05 compared with H_2_O_2_-stimulated cells. Data were from at least three independent experiments, each performed in duplicate.

Loss of mitochondrial membrane potential (ΔΨ_m_) played a pivotal role in H_2_O_2_-induced apoptosis [6]. The effect of DSC on H_2_O_2_-induced ΔΨ_m_ loss was analyzed by flow cytometry using JC-1 staining. As shown in Figure 7A, stimulation with H_2_O_2_ significantly induced the loss of ΔΨ_m_, which was attenuated by pretreatment with DSC in a concentration dependent manner. Meanwhile, cell apoptosis induced by H_2_O_2_ was also inhibited by DSC as observed by Annexin V-fluorescein isothiocyanate (FITC)/propidium iodide (PI) assay. NAC, a non-targeted ROS scavenger, also markedly inhibited H_2_O_2_-induced ΔΨ_m_ loss and apoptosis in HUVEC.

### 2.7. DSC Modulated Apoptosis-Related Proteins Expression in H_2_O_2_-Stimulated HUVEC

**Figure 8:**
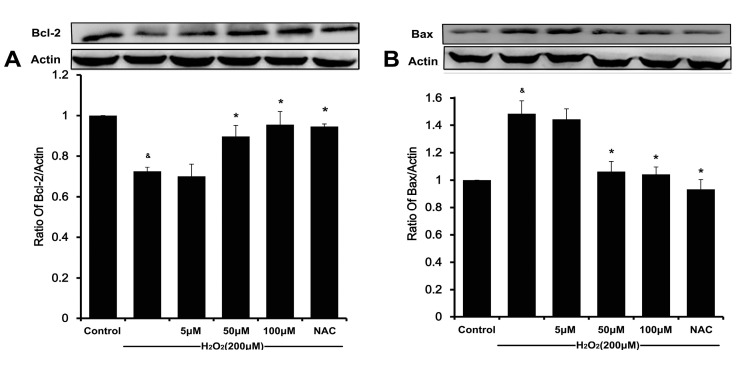

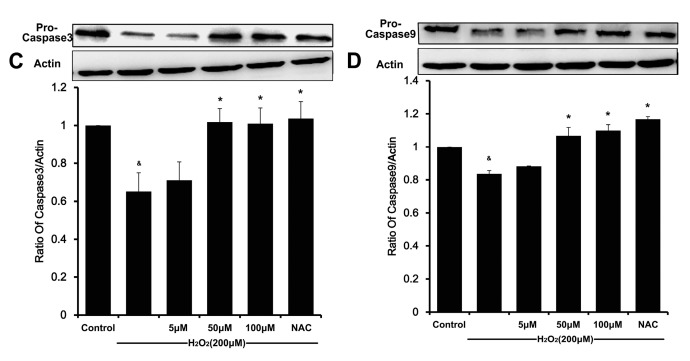
DSC modulated apoptosis-related proteins expression in H_2_O_2_-stimulated HUVEC. HUVEC were incubated with indicated concentrations of DSC or NAC for 4 h, then stimulated with H_2_O_2_ (200 μM) for 12 h; the Bcl-2 (**A**); Bax (**B**); caspase-3 (**C**); and caspase-9 (**D**) were measured by Western blot, respectively. Western blot and densitometric analysis for Bcl-2 (**A**); Bax (**B**); caspase-3 (**C**); and caspase-9 (**D**). β-actin was used as loading control. Protein expression in control cells was set to 1. Data shown are means ± SEM, ^&^ *p* < 0.05 compared with unstimulated cells, * *p* < 0.05 compared with H_2_O_2_-stimulated cells; Data were from at least three independent experiments, each performed in duplicate.

To further demonstrate the anti-apoptotic activity of DSC on H_2_O_2_-stimulated HUVEC, apoptosis-related proteins were analyzed by Western blot. As shown in Figure 8A,B, H_2_O_2_ stimulation significantly decreased Bcl-2 expression and increased Bax expression level. However, pretreatment with DSC concentration-dependently increased Bcl-2 expression and decreased Bax expression in H_2_O_2_-stimulated HUVEC. Meanwhile, H_2_O_2_ stimulation markedly induced degradation of caspase-3 and caspase-9 (Figure 8C,D). Consistent with our previous study [6], DSC concentration-dependently inhibited H_2_O_2_-induced caspase-3 and caspase-9 degradation in HUVEC. In addition, NAC treatment also attenuated H_2_O_2_-mediated changes of apoptosis-related proteins expression.

### 2.8. Discussion

Danshensu, a hydrophilic bioactive component of Danshen, attracts considerable interest due to its salubrious biological activities. However, the chemical structure of Danshensu contains unstable phenolic hydroxyl groups, which limits its further therapeutic development for clinical use [11]. Recently, we reported that the Danshensu-cysteine conjugate, DSC, is found to have an improved antioxidant and anti-apoptotic activity in *vitro* [6,12]. The present study provides the first evidence that the two diastereoisomers (**2** and **3**) of DSC are synthesized asymmetrically in excellent stereoselectivities and high yields using a chemical approach based on asymmetric hydrogenation [14]. As the presence of the active hydroxyl group contributes to the instability of Danshensu, we used the asymmetric synthesis to obtain Danshensu derivatives, with an acetyl analogue of Danshensu to eliminate the unstable hydroxyl group, which showed a much better stability in our previous study [6,12]. The cytoprotective property and the improved stability make the novel Danshensu-cysteine conjugates promising as anti-apoptotic reagents.

Recently, we demonstrated that DSC protected SH-SY5Y cells against H_2_O_2_-induced cellular injury, at least in part, due to its strong anti-oxidative and anti-apoptotic properties [6]. Therefore, the potential anti-oxidative and anti-apoptotic activity of the diastereoisomers of DSC was firstly evaluated in H_2_O_2_-stimulated SH-SY5Y cells. Consistent with previous studies [6,16], H_2_O_2_ stimulation significantly induces cellular damage and decreases intercellular anti-oxidative capacity. However, DSC and its diastereoisomers (all at 50 μM) significantly attenuate H_2_O_2_-induced cellular injury in SH-SY5Y cells, as evidenced by increase of cell viability and decrease of cellular morphological changes and nuclear condensation. However, the three compounds pretreatment also markedly increase SOD and GSH activity as well as GPx expression, and decrease LDH release and MDA production in H_2_O_2_-stimulated SH-SY5Y cells. But, the cyto-protective activity is not significantly different among these stereoisomers. These results suggest that DSC and its two diastereoisomers have similar protective effects on the anti-oxidative activity against H_2_O_2_-induced cellular damage. Our findings indicate that the Danshensu-cysteine conjugates exert their cytoprotective effects against oxidative insults through modulation of intercellular anti-oxidative capacities.

Danshen and its bioactive ingredients have been mainly used for the treatment of cardiovascular diseases [9,17]. Meanwhile, our previous studies have demonstrated that Danshensu-cysteine conjugates exhibited cardiovascular-protective activity [12,18]. Thus we hypothesize that the Danshensu-cysteine conjugates may also exert beneficial effects in cardiovascular diseases. DSC and its diastereoisomers also exert similar protective effects on H_2_O_2_-induced cellular injury in SH-SHY5Y. Taking into account the clinical application and the difficulty of chemical synthesis of the Danshensu-cysteine conjugates, DSC was chosen for further research. As expected, DSC significantly attenuates H_2_O_2_-induced cellular injury and redox imbalance in endothelial cells, which is one of the major causes of cell and tissue injury and implicated in the pathological process of cardiovascular disease [11,19]. H_2_O_2_ can induce ΔΨ_m_ disruption and release of cytochrome C from mitochondria into the cytoplasm, and therefore initiate apoptosis by activating caspase-3 [16,20]. Meanwhile, a decreasing Bcl-2/Bax ratio results in mitochondrial dysfunction, which ultimately triggers the activation of caspase cascades and results in apoptosis [6,21]. In our present study, DSC concentration-dependently reverses H_2_O_2_-mediated loss of ΔΨ_m_ and decreases the ratio of Bcl-2/Bax in HUVEC. The subsequent disruption of ΔΨ_m_ causes activating caspase-9, which in turn activates caspase-3 and eventually apoptosis [22,23]. Likewise, the degradation of caspase-9 and caspase-3 in H_2_O_2_-stimulated HUVEC was also attenuated by DSC in a concentration-dependent manner. Altogether, these results indicate that DSC protective action against cell death involves anti-apoptotic mechanisms.

## 3. Experimental Section

### 3.1. Materials and Antibodies

Starting materials and reagents were obtained from commercial suppliers and were used without purification unless otherwise stated. Melting points were determined in open capillary tubes using hot stage apparatus and were uncorrected. Flash chromatography was performed using silica gel (300–400 mesh) with the indicated solvent system. ^1^H NMR spectra were recorded at 400 MHz using tetramethylsilane as an internal standard and ^13^C NMR spectra were measured at 100 MHz with complete proton decoupling. All chemical shifts are reported in ppm on the δ scale relative to an internal standard of TMS (^1^H) or the signals of the solvent (^13^C). High-resolution mass spectra (HRMS) were recorded using either electron impact (EI) or electrospray ionization (ESI) techniques. Dulbecco’s modified Eagle’s medium/F-12 (DMEM/F-12), fetal bovine serum (FBS) were from GIBCO-BRL (Grand Island, NY, USA). NAC and 3-(4,5-dimethylthiazol)-2,5-diphenyltetrazolium bromide (MTT) were purchased from Sigma-Aldrich (St. Louis, MO, USA). Antibodies against Bcl-2, Bax, caspase-9, caspase-3, β-actin were purchased from Cell Signaling Technology (Danvers, MA, USA). All compounds (except for positive control NAC) solution in dimethyl sulfoxide (DMSO) was freshly prepared. Final DMSO concentration in media did not exceed 0.05%.

### 3.2. Cell Culture

Human neuroblastoma cells (SH-SY5Y) were grown in DMEM/F-12 containing 10% FBS, 100 U/mL penicillin and 100 μg/mL streptomycin at 37 °C in a humidified 5% CO_2_ atmosphere. Cells were exposed to different compounds for 4 h, and then subjected to 200 μM H_2_O_2_ for 12 h.

HUVEC (ATCC, Manassas, VA, USA) were maintained in DMEM containing 1800 mg/L NaHCO_3_, supplemented with 10% FBS, 1% endothelial growth supplement, 100 U/mL penicillin and 100 μg/mL streptomycin at 37 °C in a humidified atmosphere with 5% CO_2_. Passage 3–8 was used for experiments.

### 3.3. Cells Viability

Cell viability was evaluated by MTT assay as described previously [6]. In brief, MTT was added to the cells at a final concentration of 0.5 mg/mL, and cultured at 37 °C, 5% CO_2_ atmosphere for 1 h. Cells were washed with PBS and the converted formazan dye was dissolved in DMSO and measured at 570 nm by a spectrophotometer (M1000, TECAN, Austria GmbH, Untersbergstrasse, Grödig Austria).

### 3.4. Hoechst 33258 Staining

The nuclear morphological changes of apoptotic cells were detected using the Hoechst 33258 nuclear staining kit (Beyotime Biotechnology, Haimen, China) according to the manufacturer’s instructions. Nuclear morphology was observed under a fluorescence microscope (excitation, 340 nm; emission, 460 nm, Carl Zeiss Inc., Jena, Germany).

### 3.5. Annexin V-Fluorescein Isothiocyanate (FITC)/Propidium Iodide (PI) Apoptosis Assay

Early apoptosis and necrosis were identified by means of double fluorescence staining with annexin V-PI. Staining for FITC-labeled annexin V binding to membrane phosphatidylserine and PI binding for cellular DNA was performed according to the protocol provided by the manufacturer (Becton Dickinson Pharmingen, San Jose, CA, USA). Briefly, HUVEC (1 × 10^6^ cells per sample) were incubated with indicated concentrations of DSC or NAC for 4 h, then stimulated with H_2_O_2_ (200 μM) for 12 h, the cells were loaded with PI (5 µL) and annexin V-FITC (10 µL) at room temperature for 10 min in the dark and analyzed using a flow cytometer (Becton Dickinson, Franklin Lakes, NJ, USA).

### 3.6. ΔΨ_m_ Assessment

ΔΨ_m_ was determined with fluorescent probe JC-1, which exists predominantly in monomeric form in cells with depolarized mitochondria and displays fluoresced green at 490 nm. Cells with polarized mitochondria predominantly contain JC-1 in aggregate form and show fluorescence of reddish orange color. Briefly, HUVEC were incubated with indicated concentrations of DSC or NAC for 4 h, then stimulated with H_2_O_2_ (200 μM) for 2 h, after two rinses with PBS, the cells were incubated with JC-1 (2 mM) for 15 min and rinsed twice with PBS, and visualized by fluorescence microscope (Carl Zeiss) with excitation at 488 nm and emission at 520 nm or analyzed by flow cytometry (Becton Dickinson).

### 3.7. Western Blot

For whole cell extraction, cells were washed twice with ice-cold PBS and lysed in RIPA buffer with protease & phosphatase inhibitors (Sigma-Aldrich). After centrifugation (4 °C, 10 min, 10,000× *g*), samples were prepared for Western blot analysis. Western blot analyses was performed as previously described [24]. Briefly, equal amounts (30 μg) of proteins were separated and transferred to polyvinyl difluoride membrane. The membranes were probed with antibodies: Bcl-2, Bax, caspase-9, caspase-3, β-actin (all dilution in 1:1000), then incubation with either horseradish peroxidase-conjugated goat anti-rabbit antibody (1:5000, Pierce Biotechnology, Waltham, MA, USA). Immunoreactive proteins were visualized by enhanced chemiluminescence and signal intensity was detected and quantified by Alpha Imager (Alpha Innotech Corp., San Leandro, CA, USA).

### 3.8. Lactate Dehydrogenase (LDH) Release, Malondialdehyde (MDA) Production, Superoxide Dismutase (SOD) Activity, and Reduced Glutathione (GSH) Activity Measurement

The SH-SY5Y cells or HUVEC in 6-well plates were pretreated with DSC or NAC for 4 h, then stimulated with H_2_O_2_ after 12 h, LDH release, MDA production, SOD and GSH activity in the supernatants were detected by a LDH Cytotoxicity Assay Kit, Lipid Peroxidation MDA Assay Kit, Total Superoxide Dismutase Assay Kit, and GSH/GSSG Assay Kit Assay Kit (all from Beyotime Biotechnology, Haimen, China) according to the manufacturer’s instructions, respectively.

### 3.9. Immunofluorescence

SH-SY5Y cells were grown on glass slides in six-well plates. Cells were fixed in 4% paraformaldehyde for 30 min at room temperature, immunostained using rabbit anti-GPx (all 1:200) and Alexa Fluor 488-conjugated goat anti-rabbit IgG (1:500; Invitrogen, Carlsbad, CA, USA). Immunofluorescence was visualized using a fluorescence microscope (Carl Zeiss). The results were based on three independent analyses.

### 3.10. Statistical Analysis

Data are reported as mean ± SEM. Differences between mean values of multiple groups were analyzed by one-way analysis of variance with Tukey’s test for *post hoc* comparisons. The level of significance was determined at *p* < 0.05.

## 4. Conclusions

In summary, this study for the first time reports using the method of asymmetric synthesis to obtain the two diastereoisomers of DSC. Our present experiments also indicate that DSC and its two diastereoisomers have similar bioactivity on H_2_O_2_-induced cellular damage. Furthermore, our results demonstrate that DSC protective action against cell death involves anti-apoptotic mechanisms in endothelial cells. These results suggest that DSC possesses cytoprotective effects and that DSC supplementation may be a promising therapeutic strategy for oxidative stress-related diseases.

## Abbreviation

ΔΨ_m_: mitochondrial membrane potential
DIPEA: diisopropylethylamine
DMEM/F-12: Dulbecco’s modified Eagle’s medium/F-12
DMSO: dimethyl sulfoxide
DSC: *N*-((*R*)-3-benzylthio-1-methoxy-1-oxo-2-propanyl)-2-acetoxy-3-(3,4-diacetoxyphenyl) propanamide
EDCI: 1-(3-dimethylaminopropyl)-3-ethylcarbodiimide hydrochloride
EI: electron ionization
ESI: electrospray ionization
FBS: fetal bovine serum
HOBt: 1-hydroxybenzotriazole
GPx: glutathione peroxidase
GSH: reduced glutathione
H_2_O_2_: hydrogen peroxide
HUVEC: human umbilical vein endothelial cells
LDH: lactate dehydrogenase
MDA: malondialdehyde
MTT: 3-(4,5-dimethylthiazol)-2,5-diphenyltetrazolium bromide
NAC: *N*-acetyl-l-cysteine
PI: propidium iodide
SOD: superoxide dismutase
TMS: tetramethylsilane
